# Evaluation of a pressure head and pressure zones in water distribution systems by artificial neural networks

**DOI:** 10.1007/s00521-017-2844-8

**Published:** 2017-01-12

**Authors:** Jacek Dawidowicz

**Affiliations:** Faculty of Civil and Environmental Engineering, Bialystok University of Technology, ul. Wiejska 45A, Białystok, Poland

**Keywords:** Water distribution system, Hydraulic calculations, Pressure zones, Multilayer perceptron, Neural classification

## Abstract

Water distribution system design is inherently associated with hydraulic calculations that require thorough evaluation of obtained results and accuracy of the applied solution. Currently, there are no programs that will replace a designer in these tasks, and there likely will not be such programs. However, some individuals are trying to develop computer programs featuring a certain degree of creativity to facilitate user decision making. In water distribution system design and hydraulic calculations, one should, inter alia, check pressure heads in different parts of the system. It is also important to establish whether the system should contain one or more pressure zones. This determination is connected with the appropriate location of booster and pressure reducing stations. In this paper, the nominal variable is defined. The classes of this variable describe problems related to a value of pressure and division of the water distribution system into pressure zones. By choosing one of the classes, an artificial neural network determines the problems that may arise in a given part of the water distribution system. The classification is conducted based on neural network input variables describing the specific parameters that affect water distribution system design, such as land relief, loss of pressure, pipe roughness and distance to a water supply. The paper presents a new approach that extends traditional methods of hydraulic calculations for water distribution systems by introducing the evaluation of a pressure head and the analysis of design concepts of pressure zones by using artificial neural networks.

## Introduction

Water distribution system design is inherently associated with hydraulic calculations. The first specialized computer programs emerged in the 1960s [1, 2]; since then, there has been noticeable progress in technology and features offered by the latest water distribution system calculation programs [3] that apply GIS [4, 5] and CAD [6] to a wide extent. However, this does not mean that execution of hydraulic calculations does not require thorough evaluation of results and solution accuracy. Currently, there are no, and there likely will not be, programs that can replace a designer in these tasks. We try to develop computer programs with a degree of creativity in order to facilitate user decision making at given stages of task realization and improve the quality of solutions. Traditional algorithms can be complemented with more advanced calculation techniques known as computing intelligence methods [7, 8]. This approach also involves a method of modeling by artificial neural networks [9, 10].

Artificial neural networks are widely applied to solve technical problems. A review of the application of neural networks in the water supply area is provided by a report prepared by the American Society of Civil Engineers [11]. This review covers a wide range of problems and addresses, inter alia, the issue of function approximation in water distribution system modeling [12]. Saldarriaga et al. [13] and Lingireddy and Ormsbee [14] suggested using artificial neural networks to support tarring of simulation models for water distribution systems. Shayya and Sablani [15] present a non-iterative method of friction factor calculation for the Darcy–Weisbach formula to calculate pressure loss along water supply lines. Calculation modules based on artificial neural networks were introduced as simulation methods applied to control water supply networks in real time [16, 17]. In this case, the goal of calculations using neural networks is to simplify and accelerate simulation modeling. Monuce et al. [18, 19], Monuce and Machell [19] and Zhu et al. [20] propose a method for detection and localization of bursts in pipelines using artificial neural networks. Mansoor and Vairavamoorthy [21] discussed a modified water supply system model in which water consumption by users at a node depends on the water pressure head in a network. The purpose of the modifications is to enable evaluation of water consumption in case of a network failure and pressure drop. Arsene et al. [22] presented a multilevel neural network-based decision support system (DSS) for regular water distribution system monitoring and control. System monitoring and operational checks include pipeline leak detection.

Summing up analysis of the literature, artificial neural networks have practical applications in problems concerning water supplies; however, the evaluation of a pressure head and zoning system, location of booster pumps or pressure reductions have not been examined. This paper reports on the author’s study to establish whether artificial neural networks can be used in solving the above problems. It complements the traditional hydraulic calculation methods for water distribution systems with artificial networks.

## Numerical modeling of water distribution systems

There are two versions of numerical water distribution system modeling: extended period analysis [3, 6, 23] and steady-state analysis [24, 25]. In the extended period analysis, computations are carried out over an extended period of time at variable water consumption and variable system operating conditions. The steady-state analysis is carried out for defined water consumption conditions and system operating conditions. In this paper, steady-state analysis was applied for peak hour water demand (PHD). The above conditions are the basis for dimensioning the water distribution system elements and determination of the number of pressure zones in a system. There are also attempts to use the proposed method of assessing the pressure and zoning of the water distribution system in simulations over a longer period of time for a maximum water intake hour.

To carry out calculations, the water distribution system is represented by graph theory [26, 27]. A system is understood as a set of graph branches, corresponding to water pipes and nodes, describing pipe connections and endings. Nodes in a model can represent points of water supply, distribution or storage. The following parameters are ascribed to nodes: terrain coordinates, nodal water distribution, required pressure head or current pressure head. Pipes are defined by parameters such as length, diameter, flow rate, velocity and roughness coefficient. System elements such as pump stations and valves are represented by graph branches.

A hydraulic model of a distribution system is constructed as a system of nonlinear equations whose structure is based on the mass balance laws in nodes and energy equilibrium in loops also called the Kirchhoff laws [25, 28]. Several methods can be used to solve the above model. The first of these was developed in 1936 by Hardy Cross [29]. In 1963, Martin and Peters used the Newton–Raphson method for this purpose [30]. Successive work resulted in the gradient method [31] and the hybrid method [32]. Todini [33] presented the characteristics of five algorithms based on the Newton–Raphson technique that differ in the variables used for nonlinear equation system solving a water distribution system model. These methods enable hydraulic calculations of a water distribution system of any structure and size; however, they do not comprise elements supporting decisions or analysis of obtained results.

## Problems with pressure head and pressure zoning in water distribution system design and calculation

Water distribution system design requires hydraulic calculations to determine flow rates through individual system sections, pipe diameter selection, pressure loss calculation and pressure head value determination at nodes. The calculations require thorough verification of the results. The most important criteria used to evaluate water distribution system performance include the water flow velocity through pipes and the pressure head in nodes. In this paper we deal with the problem of evaluation of a pressure head and related pressure zoning.

Pressure head (PH) is the height of a column of water necessary to develop a specific pressure at a given point. Proper values of a pressure head in an entire water distribution system should ensure the pressure head at individual nodes is between the required pressure head *H*_r_ and the maximum pressure head *H*_max_.

Determining whether the current pressure head in a network node has a value within the recommended range is relatively simple. Algorithms used in the programs allow calculating pressure losses in individual pipes and a pressure head in nodes but do not analyze the pressure head in an entire water distribution system. An incorrect value of the pressure in a node can be caused by various reasons. It is important for a computer program to be able to point to an incorrect value during hydraulic calculations, diagnose a cause and possibly suggest a solution that will solve the problem connected with the assessment of the pressure value in nodes of the water distribution system.

## Artificial neural networks

Artificial neural networks (ANN) are a technique of modeling enabling representation of complex, multidimensional and nonlinear relationships between inputs and outputs. In this paper the most popular neural network architecture, the multilayer perceptron (MLP), was applied [9, 10, 34].

The most desirable property of a neural network is its capability to generalize and translate its knowledge into new cases. To improve the neural network generalization cross-validation can be applied. For this purpose, all cases were divided into three parts: a training subset, a validation subset and a test subset.

The problem of multi-class classification was investigated by the MLP neural network. Classification is carried out by the nominal output variable, with the nominal values corresponding to various classes. The purpose of the neural network is to assign each input data *X* = [*x*_1_, *x*_2_,…, *x*_*n*_]^T^ to one of the number of classes. If the MLP network is used in multi-class classification problems, the Softmax activation function should be applied in the output layer [35]. It is a function whose result is standardized to obtain the sum of activation of all m neurons of the output layer equal to 1. The output signals from the network serve as a basis to recognize an appropriate class, and the activation values from individual neurons can be interpreted as a probability estimation of assignment of an output signal to a given class.

The neural network described in this paper was trained with regard to the multiple cross-entropy error function. This error function is specially designed for classification problems concerning multiple-output networks [9, 36].

## Development of the model of an artificial neural network for the assessment of a pressure head and division of the water distribution system into pressure zones

### Data collection

The application of the method with artificial neural networks requires the preparation of a set of training data. The set has to include training cases representing input and output variables within the scope of the variability under investigation. In this paper, the set of cases was obtained on the basis of the results of calculations for water distribution systems for the time-point at the peak water distribution. The hydraulic calculations were based on the data from eight existing water distribution systems. In addition, the basic variants were modified in order to obtain systems that require pressure zoning. For different pressure conditions, network pump stations or pressure reducers were applied. Thus, the training set includes cases requiring zoning and the application of the above devices. Some variants of the calculations covered water distribution systems with incorrectly sized pipe diameters; however, training examples represent proper conditions as well as conditions calling for a correction of the design of a water distribution system.

The problem of the evaluation of a water distribution system was treated as a classification task. For this purpose, the nominal variable CWDS (class of water distribution system) was defined, and it comprises five classes describing problems resulting from improper pressure head values and one class related to the correct hydraulic grade line course from the selected water distribution node to the water supply node. The analysis was carried out for each node separately; hence, the corresponding class was assigned subsequently to a given node in the water distribution system by the artificial neural network. Classes and their corresponding labels are defined as follows:

- pressure head at a given water distribution node above the maximum value resulted from too high a pressure head at the water supply node (label PHA—Pressure Head Above),
- pressure head at a given node below the required value resulted from too low a pressure head water at the supply node (label PHB—Pressure Head Below),
- recommended pipe diameter correction in order to reduce pressure losses between the water supply node and a given water distribution system node (label RDC—Recommended Diameter Correction),
- recommended installation of a network pumping station in a given node and separation behind a given node of a separated pressure zone (label NPS—Network Pumping Station),
- recommended installation of a pressure reducer at a given node and separation after a given node of a separated pressure zone (label PRD—Pressure Reducer),
- proper pressure head and hydraulic grade line between the water supply node and a selected water distribution system node (label PHGL—Proper Hydraulic Grade Line).

Assignment of a given class to a node depends on parameters describing land relief, pipe length from the water supply node to individual water distribution system nodes, a water supply pressure head and a pressure head at individual nodes, pressure losses and roughness height on the inside walls of pipes. To precisely describe the parameters characterizing the class of the CWDS variable, the components of the input vector *X* for artificial neural network learning are defined as follows:

- *x*_1_ pressure head at the water supply node,
- *x*_2_ length of the shortest distance in a network from the supply node to a selected water distribution system node,
- *x*_3_ difference of land levels between the supply node and a selected node,
- *x*_4_ largest difference in land levels along the shortest distance between the supply node and a selected node,
- *x*_5_ sum of pressure losses along the shortest distance between the supply node and a selected water distribution system node,
- *x*_6_ highest pressure head along the shortest distance between the supply node and a selected water distribution system node,
- *x*_7_ weighted average of absolute roughness k of pipelines along the route between the supply node and a selected system node,
- *x*_8_ pressure head at a selected system node.

**Fig. 1:**
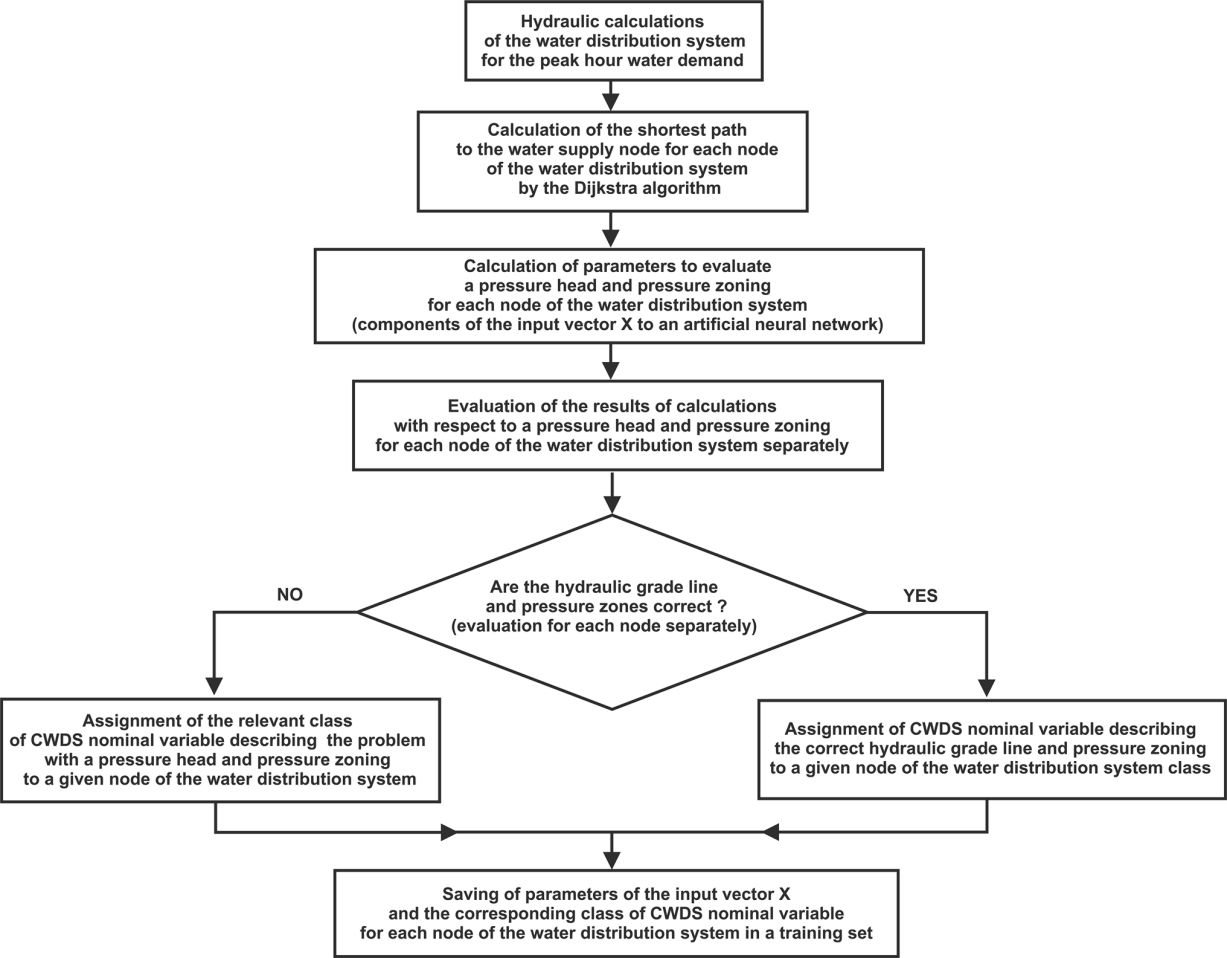
Diagram of data gathering for training set

According to the supervised learning, an appropriate class of the CWDS variable was assigned to every node after calculation of the input vector *X* components corresponding to them. Preparation of the training set followed the principle that water in a water distribution system should be distributed using the shortest distance from the supply source to a user. To calculate the vector *X* components, a numerical procedure was developed that carried out computation of individual vector *X* components for all water distribution system nodes supplied from the supply source and saved the values obtained in the training data set. The shortest distance between the supply source and successive nodes of a water distribution system was calculated with the Dijkstra algorithm [37]. Finally, a set of 8427 of training cases was obtained that represented all the classes. Figure 1 diagrams the data collection process for the training set.

### Design of a neural network model

Neural networks can have any number of layers and any number of neurons in each layer. The neuron number in the input and output layers is determined by the problem the model solves. The main problem in designing neural networks is the choice of the number of hidden layers and the number of neurons in each of the layers. Another aspect is the selection of the activation function and the error function. To determine the initial number of neurons in hidden layers apply a formula proposed by Hecht-Nielsen [38]:

1 $$L = 2N + 1$$

where *L* is the number of neurons in the hidden layer and *N* is the number of input neurons.

The search for the most advantageous neural network architecture is carried out by increasing the number of neurons in the hidden layers and the number of layers. It should be remembered that increasing the number of neurons in the hidden layers can lead to the problem of over-fitting.

Training of the multilayer perceptron of different architectures resulted in adoption of the neural network, which consisted of the following elements:

- input layer with 8 neurons, corresponding to the components of the input vector *X*,
- first hidden layer consisting of 85 neurons with the logistic activation function,
- second hidden layer consisting of 68 neurons with the logistic activation function,
- output layer of 6 neurons with the Softmax activation function where the neurons correspond to the components of the CWDS variable.

**Fig. 2:**
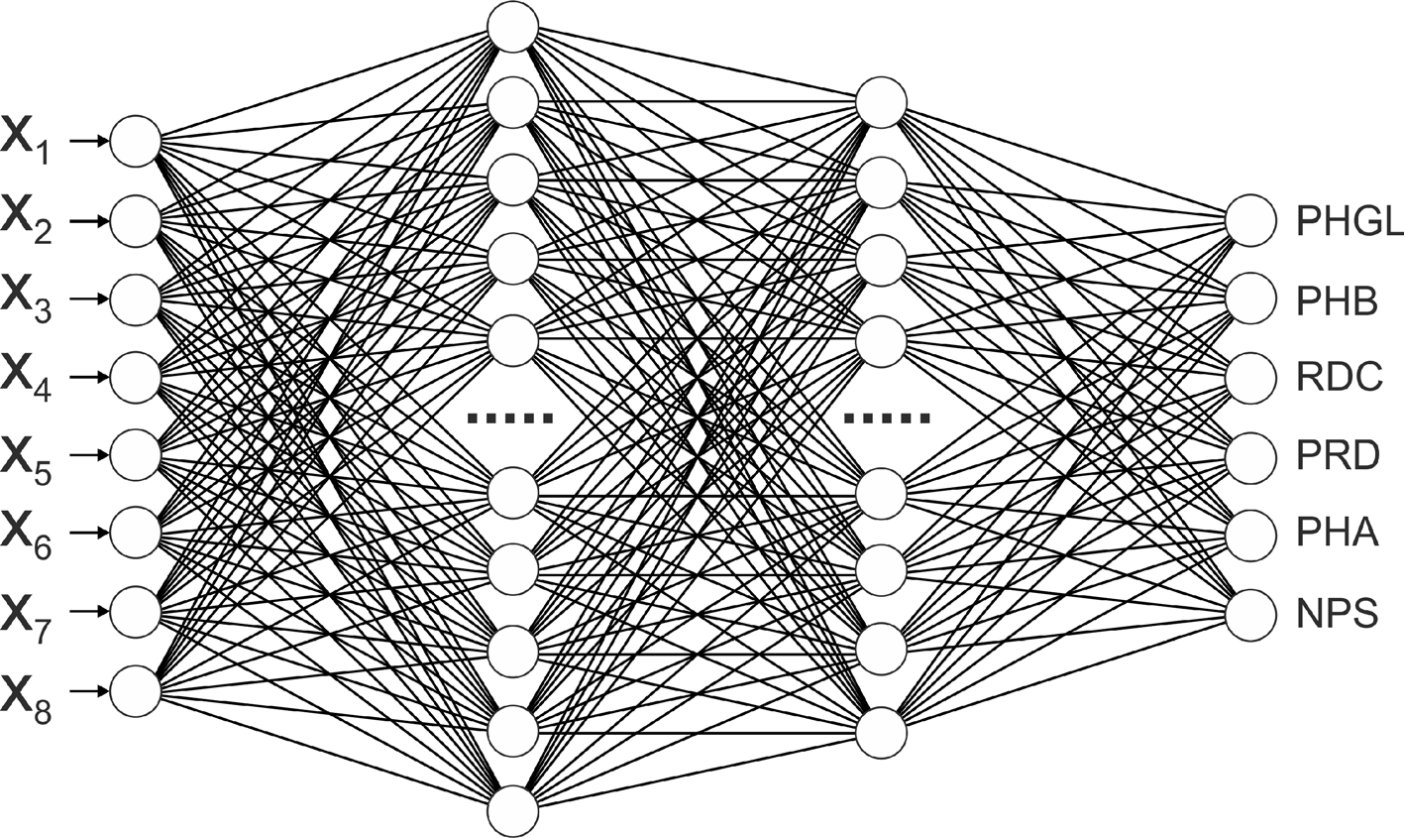
Scheme of the neural network for evaluation of a pressure head and pressure zones in water distribution systems

The scheme of the artificial neural network for the CWDS variable component classification is presented in Fig. 2.

Due to the application of the Softmax activation function in the output layer for the unclassified cases, the probability of assignment of every case to individual classes could be evaluated. The detailed evaluation of the classification results by the obtained neural network for individual subsets of cases is presented by the confusion matrix. It is a square matrix in which information on assignment to each class is given in lines and information on a class in which an individual example is classified by the classifier is given in columns. Correctly classified examples are located on the diagonal, and improperly classified examples are outside of the diagonal. Examples located outside the diagonal indicate which class was incorrectly ascribed to them. The top of the table summarizes overall statistics: the total number of cases of each class in the data set and the number of cases classified as correct, incorrect and unknown.

Table 1 presents the classification results for the training subset, Table 2 for the validation subset and Table 3 for the test subset. The accuracy classification is 0.958, 0.945 and 0.938 for the training, validation and testing subsets, respectively. The results presented in the tables for the training, validation and test subsets indicate that the developed MLP network can correctly predict the class variable CWDS. Accordingly, the neural network is applied to develop methods for assessing the pressure head and pressure zoning in water distribution systems.

**Table 1:** Classification results for the training subset

	PHGL	PHB	RDC	PRD	PHA	NPS
Total	923	513	647	735	723	752
Correct	860	481	616	725	651	736
Incorrect	7	0	0	1	6	0
Unknown	56	32	31	9	66	16
PHGL	860	0	0	1	1	0
PHB	2	481	0	0	0	0
RDC	1	0	616	0	4	0
PRD	1	0	0	725	0	0
PHA	3	0	0	0	651	0
NPS	0	0	0	0	1	736

**Table 2:** Classification results for the validation subset

	PHGL	PHB	RDC	PRD	PHA	NPS
Total	471	268	285	373	370	377
Correct	438	258	274	366	320	370
Incorrect	0	0	0	4	7	0
Unknown	33	10	11	3	43	7
PHGL	438	0	0	3	2	0
PHB	0	258	0	0	0	0
RDC	0	0	274	1	1	0
PRD	0	0	0	366	0	0
PHA	0	0	0	0	320	0
NPS	0	0	0	0	4	370

**Table 3:** Classification results for the testing subset

	PHGL	PHB	RDC	PRD	PHA	NPS
Total	447	274	316	380	363	364
Correct	407	258	297	374	318	358
Incorrect	8	0	1	4	9	0
Unknown	32	16	18	2	36	6
PHGL	407	0	0	2	2	0
PHB	0	258	0	0	0	0
RDC	3	0	297	2	2	0
PRD	2	0	0	374	0	0
PHA	3	0	1	0	318	0
NPS	0	0	0	0	5	358

The results presented in Tables 1, 2 and 3 indicate a very high level of classification. Incorrect results can be further evaluated by the value of the activation of neurons in the output layer.

## Method of evaluation of a pressure head and pressure zones for a water distribution system

The analysis of a system of pressure zones is based on the assumption that computer calculations for a water distribution system are a process that requires iterative calculations to achieve an appropriate solution. Therefore, “correct process status” (CPS) is adopted to end the calculation process, and the “incorrect process status” (IPS) requires the solution to be corrected and calculations to be repeated. The process status is determined on the basis of classification results by means of the artificial neural network. During the analysis, the CWDS variable components are generated as an additional parameter for all water distribution system nodes that helps to establish whether the pressure head is within acceptable limits and whether the system requires an additional pressure zone. When the pressure does not fall within the required values, the appropriate component of the CWDS variable suggests a solution of the problem, e.g., if a network pumping station or a pressure reducer should be mounted on a given node. In case the NPS component is assigned to a given node that suggests mounting of a pumping station, or the PRD component signifies installation of a pressure reducer, further analysis is required after a given node is interrupted in order to modify the system structure. The activation value of a neuron from the output layer of the multilayer perceptron with the Softmax activation function is ascribed to every node. Thus, the classification certainty of the CWDS component can be estimated. The detailed analysis of nodes that have not been assigned a class is required, but because of the high quality of the classification neural network, these should be rather low. For these nodes, the class is determined by a person executing the water distribution system calculation.

The classification is conducted on the basis of the defined vector *X* components calculated for every system node. With reference to the above, the method of the water distribution system analysis consists of the following stages:

- hydraulic calculations for the peak hour water demand,
- calculations of the vector *X* components of input variables of the neural network for all nodes,
- classification of the CWDS variable rated component for all nodes,
- analysis of the activation value of neural network output layer neurons for all nodes,
- analysis of the nodes where no class of the CWDS variable was automatically assigned,
- determination of the process status (CPS or IPS),
- if the correct process status (CPS) is achieved—completion of calculations,
- in case of final incorrect process status (IPS)—correction of the design and further hydraulic calculations.

The correct status of the process—CPS—is regarded as achieved at the moment when the correct pressure head is obtained at all nodes, i.e., the PHGL component of the CWDS variable is assigned to these nodes. Otherwise, the incorrect process status—IPS—is regarded as achieved, and consequently, there is a need to change the arrangement of pressure zones, alter the pipe diameters or possibly change the supply pressure.

## Conclusions

There are many methods of calculation of water distribution systems that enable solving tasks for various structures of pipeline. Computer programs based on the above methods calculate the value required for variables describing the individual elements of the system, but do not have procedures for assessing their accuracy. Due to increasing requirements for quality of the hydraulic system calculation, professionals strive to find new methods to rationalize the calculation process and to introduce intelligent support elements. This paper presents the artificial neural network and its application for the analysis of a pressure head and pressure zones in a water distribution system.

To find the most advantageous structure of the multilayer perceptron, the training of 7 networks with one hidden layer and 9 networks with two hidden layers was carried out. In the case of a single-layer network, the search for an appropriate structure was started on the basis of the Hecht-Nielsen formula, from 17 neurons in the hidden layer, by increasing in each subsequent network from the initial value to 119 neurons. Single-layer networks were characterized by a low capacity of classification of components for the CWDS variable. That is why the training of two-layer networks was initiated. Two-layer networks were trained from a structure of 8-17-17-6, successively increasing the number of neurons in the first hidden layer with the initial value 8-34-17-6, then in the second layer 8-34-34-6 and ending in the structure 8-85-85-6. More complex structures of two-layer networks were very susceptible to the phenomenon of over-fitting. The multiple cross-entropy error function for a subset of validation was used to assess progress of training and select the most favorable structure. To avoid local minima, each structure of the neural network was trained repeatedly, and then, the most favorable option was retained. For the initial two-layer network 8-17-17-6, the final value of the error function amounted to 0.6265376 for the training subset and 0.6374514 for the validation subset, while for the adopted structure 0-85-68-6 it amounted to 0.2084361 for the training subset and 0.2408573 for the validation subset. The analysis of sensitivity of the input variables was carried out, but all variables showed a significant impact on the error in teaching the neural network. The resignation of any variable resulted in a significant increase in a teaching error.

On the basis of the obtained results, one can conclude that the method presented can complement traditional calculation methods. Using a neural network of a proper structure, the components of the CWDS variable can be classified very precisely. An additional asset is that the Softmax function can be applied in the outlet neural layer allowing for the probability estimation of adopting a given solution.

Water distribution systems are often evaluated many times for each variant; as a rule, the calculation does not give a correct solution after the first pass. Therefore, it can be assumed that this is a multi-stage computational process. Further scientific work will attempt to implement elements of the diagnostics of processes in calculations of the water distribution system neutral model. The use of neural modeling techniques in combination with elements of the diagnostics of processes should translate into an improvement in the functioning of water supply systems, reduction in operational costs and improvement in the conditions of service and exploitation.
